# 224. Evaluating the Epidemiology of Bloodstream Infections: A Population-Based Study

**DOI:** 10.1093/ofid/ofab466.426

**Published:** 2021-12-04

**Authors:** Elaha Niazi, Kwadwo Mponponsuo, Ranjani Somayaji, Elissa Rennert-May, John Conly, Dan Gregson, Jenine Leal

**Affiliations:** University of Calgary, Calgary, Alberta, Canada

## Abstract

**Background:**

Bloodstream infections (BSI) are a major cause of morbidity, mortality, and health care costs worldwide. Population-based studies are key to assess BSI epidemiology over time while minimizing selection bias but remain limited. Therefore, we aimed to assess the incidence of BSI in a large Canadian health region in a contemporary period. We hypothesized that there would be significant age and sex-based differences including over time.

**Methods:**

We conducted a retrospective cohort study from 2011 through 2018 using a population-based microbiology database to determine the annual age- and sex-specific BSI testing and case rates with the census as the population reference. BSI was defined as a positive blood culture for a pathogen. Episodes > 30 days apart were included for analysis. Incidence rate ratios (IRR) for testing and case rates including by sex were calculated to assess changes over time. All analyses were run at a two-sided α of 0.05 and were conducted with R 4.0.4.

**Results:**

A total of 154,147 distinct individuals (49.9% male) were analyzed and 22,869 (14.8%) had a BSI at the first encounter in the study period. Overall BSI testing incidence ranged from 1529 to 1707 per 100,000 person-years and case incidence ranged from 180 to 292 per 100,000 person-years. Testing and case incidence for BSI was greatest in the 0-4 and 75+ years age groups (p < 0.01). Males compared to females had greater testing and case incidence rates in young and old age groups, but females had greater rates in the 15-44 years groups (p < 0.01). Overall IRR for cases comparing 2018 to 2011 was 0.62 (95% CI 0.59-0.65) reflecting a significant decrease over time. Testing also decreased over the study period with an IRR of 0.90 (95% CI 0.88-0.91). Testing and case IRRs were not significantly different stratified by sex.

Incidence rates (per 100,000 person-years) of BSI testing and cases by sex from 2011 through 2018 in a Canadian health region

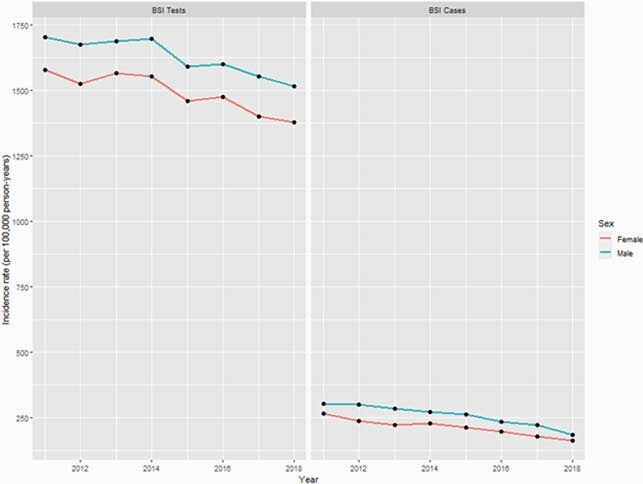

**Conclusion:**

In our large population-based study of BSI, we identified that BSI remain frequent and the youngest and oldest age groups as well as males in these age groups have the greatest BSI incidence rates which may reflect both biological sex and gender-based differences. Encouragingly, BSI incidence rates have decreased over time at a greater increment relative to testing rates. Future studies of BSI should focus on pathogen and outcome-based evaluations.

**Disclosures:**

**All Authors**: No reported disclosures

